# Directed donations for unvaccinated blood: A departure from evidence‐based medicine associated with clinical harm, resource waste, and oversight gaps in a two‐year single‐center series

**DOI:** 10.1111/trf.70195

**Published:** 2026-03-28

**Authors:** Jeremy W. Jacobs, Erika Hall, Toufik Tahiri, Princess Taylor, Chaitali Patel, Miriam Brown, Kaycie Atchison, Angela Mueller, Deva Sharma, Garrett S. Booth

**Affiliations:** ^1^ Department of Pathology, Microbiology, and Immunology Vanderbilt University Medical Center Nashville Tennessee USA; ^2^ Quality, Safety, & Risk Prevention Vanderbilt University Medical Center Nashville Tennessee USA; ^3^ Division of Hematology/Oncology, Department of Medicine Vanderbilt University Medical Center Nashville Tennessee USA

**Keywords:** blood safety, COVID‐19 vaccination, directed donation, ethics, refusal of care, transfusion service operations

## Abstract

**Background and Objectives:**

Requests for “unvaccinated” blood have been discouraged by professional and regulatory bodies because they lack scientific support and may negatively impact patient care. We describe a single‐center series in which patients or surrogates refused standard blood components unless sourced from directed donors perceived to be “unvaccinated.”

**Materials and Methods:**

We performed a retrospective review of directed donations received at Vanderbilt University Medical Center between January 1, 2024 and December 31, 2025. Data included demographics, clinical scenario, component details, and documented operational or clinical consequences.

**Results:**

Directed donor units were received for 15 patients; 13 of these patients were transfused at least one unit (*n* = 31 directed components: 22 red blood cell [RBC] units, 5 platelet units, 2 plasma units, 2 cryoprecipitate units). Median age was 17 years (range 0.33–73); nine patients were pediatric (<18 years). An ethics consultation was documented for one case, and a transfusion medicine consultation was documented for one case. Seven patients (47%) had at least one directed unit that was not transfused to them. Two patients clinically deteriorated in the setting of refusal of standard components, one of which also received a transfusion that deviated from institutional transfusion guidelines to avoid outdating, and two additional patients had surgical delay/cancellation with rescheduling associated with directed component availability.

**Conclusion:**

In this series, all directed units were from “unvaccinated” donors. These requests were associated with care delays, escalation, and inefficiencies. Health systems should implement standardized counseling, documentation, and escalation pathways consistent with existing guidance.

**Abbreviations**
HLA: human leukocyte antigen
RBC: red blood cell
TA‐GVHD: transfusion‐associated graft‐versus‐host disease
TRALI: transfusion‐related acute lung injury
VUMC: Vanderbilt University Medical Center

## 1 INTRODUCTION

In the United States and other high‐income countries, the safety and availability of the blood supply depend fundamentally on anonymous, voluntary, nonremunerated blood donation.^1^, ^2^ This model is based on the principles of altruism and community solidarity and has proven highly effective in maintaining blood safety through rigorous donor screening, infectious disease testing, and quality oversight.^2^

Directed donation—where blood is collected from a specific donor for a designated recipient—is infrequent^3^, ^4^ but serves important medical purposes in select circumstances, such as patients with rare blood types lacking compatible community donors.^5^, ^6^, ^7^ Professional guidance emphasizes that directed donation should be reserved for such medical indications,^8^, ^9^, ^10^, ^11^ though its use has not always been confined to them.

Non‐medically indicated directed donation first gained prominence during the early HIV/AIDS epidemic, when public anxiety about transfusion‐transmitted infection led patients and families to seek blood from known donors perceived as lower risk.^12^ Subsequent advances in donor screening and nucleic acid testing have rendered the volunteer blood supply extraordinarily safe, with residual per‐unit infectious risk estimated at <1 in 1 million for major transfusion‐transmitted viruses,^13^, ^14^, ^15^, ^16^ largely obviating the fear‐based rationale that originally popularized the practice.

Despite these safety advances, the COVID‐19 pandemic has prompted a resurgence of fear‐driven directed donation requests. Requests for blood from “unvaccinated” donors have emerged as a recurring challenge for transfusion services and clinicians, prompted primarily by misinformation about blood safety from vaccinated donors.^12^, ^17^, ^18^, ^19^, ^20^, ^21^ Recent legislative proposals in multiple US states have sought to mandate such accommodations.^18^, ^20^ Regulatory and professional organizations have opposed these non‐evidence‐based policies, emphasizing that blood centers do not record or convey donor COVID‐19 vaccination status and that evidence demonstrates transfusion from vaccinated donors poses no unique risk.^8^, ^10^, ^11^, ^22^, ^23^, ^24^

Because vaccination status cannot be verified through standard blood supply channels, patients seeking “unvaccinated” blood have increasingly resorted to directed donation from known donors. Unlike medically indicated directed donations, these requests reflect concerns about donor characteristics unrelated to transfusion safety. Moreover, directed donations demonstrate higher rates of infectious disease marker reactivity compared with repeat community donors, particularly among first‐time parental donors.^25^, ^26^ While prior publications have outlined the theoretical impacts of such requests on patients and the healthcare system,^5^, ^6^, ^19^ data documenting real‐world consequences when requests proceed without clinical oversight remain limited.

We describe a 2‐year, single‐center series of directed donations where the documented rationale was refusal of standard inventory due to “vaccinated blood” concerns, focusing on clinical outcomes, component utilization, and downstream operational consequences.

## 2 MATERIALS AND METHODS

We conducted a retrospective observational study of all patients for whom ≥1 directed donor unit was received in the Vanderbilt University Medical Center (VUMC) blood bank between January 1, 2024 and December 31, 2025. For each included patient, we abstracted demographics, clinical context (e.g., inpatient/outpatient status and elective vs. urgent/emergent circumstances), and the documented reason for directed donation. We reviewed transfusion service notes and the electronic health record for evidence of transfusion medicine involvement and/or ethics consultation, as well as downstream impacts when documented, including procedural delay/cancellation and/or clinical deterioration associated with awaiting directed products, and directed components collected for a patient but not transfused to the intended recipient (reflecting resource inefficiency). For each directed component, we recorded component type, quantity, and final disposition: transfused to the intended recipient, released to general inventory after conversion, retained in frozen inventory, or discarded/expired. This study was approved by the Vanderbilt University Medical Center Institutional Review Board.

Directed donation requests within our health system are frequently initiated by clinical teams and/or families through external, independent blood donor center workflows, and pre‐collection counseling may occur outside documentation visible to the transfusion service. Accordingly, this study does not estimate the overall frequency of directed donation requests that did not result in unit collection and delivery to VUMC; rather, it characterizes episodes observable through blood bank receipt and issuance records.

## 3 RESULTS

**FIGURE 1:**
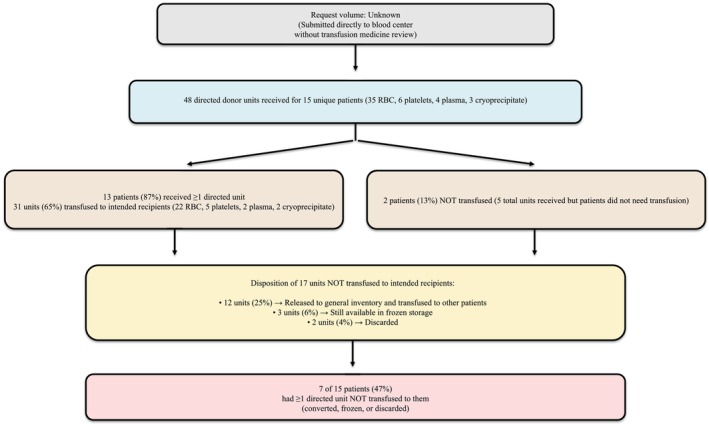
Flow chart of directed donor unit disposition.

During the study period, the VUMC blood bank received 144,856 total blood product units, of which directed donor units accounted for 0.03% (48/144,856). These 48 directed donor units were received for 15 patients, and in all cases, the request was motivated by concerns about blood from vaccinated donors. No directed donor units were received for other indications, such as rare blood types. The directed units consisted of 35 RBC units, six platelet units, four plasma units, and three cryoprecipitate units (Figure 1). Thirteen patients (87%) were transfused with at least one directed unit; two patients (13%) were not transfused despite unit arrival, accounting for 5 units (all RBCs). There was no documented evidence of poor outcomes due to non‐transfusion in these two patients. The number of patients for whom directed units were received in the blood bank increased over time, from four patients in 2024 to 11 in 2025.

Among the 48 units received, 31 (65%) were transfused to their intended recipients: 22 RBC, 5 platelets, 2 plasma, and 2 cryoprecipitate (Table 1). The remaining 17 units (35%) had alternative dispositions: 12 (25%) were released to general inventory and transfused (12 RBCs), 3 (6%) remain in frozen storage for the directed recipient (2 plasma and 1 cryoprecipitate), and 2 (4%) were discarded (1 platelet and 1 RBC) due to outdating. Notably, 7 of the 15 patients (47%), including the 2 who were not transfused, had at least 1 unit collected that was not transfused to them (Table 2).

**TABLE 1:** Directed unit utilization summary and disposition.

Component	Units received	Transfused to intended recipient	Released to general inventory and transfused to other patients	Still available in frozen storage	Discarded
RBCs	35	22	12	0	1
Platelets	6	5	0	0	1
Plasma	4	2	0	2	0
Cryoprecipitate	3	2	0	1	0
Total	48	31	12	3	2

**TABLE 2:** Outcomes stratified by age group.

Outcome	Pediatric (<18 y), *n* = 9	Adult (≥18 y), *n* = 6	All patients, *n* = 15
Directed units received	31	17	48
Units transfused to intended recipient	18 (58%)	13 (76%)	31 (65%)
Units not transfused to intended recipient	13 (42%)	4 (24%)	17 (35%)
Patients with ≥1 unit not transfused to them	5/9 (56%)	2/6 (33%)	7/15 (47%)
Patients transfused ≥1 directed unit	7/9 (78%)	6/6 (100%)	13/15 (87%)
Patients with no directed units transfused to them^a^	1/9 (11%)	1/6 (17%)	2/15 (13%)
Clinical deterioration while awaiting directed units	1/9 (11%)^b^	1/6 (17%)^c^	2/15 (13%)
Transfusion deviating from institutional guidelines	1/9 (11%)^b^	0/6 (0%)	1/15 (7%)
Surgical delay or cancellation	2/9 (22%)^d^	0/6 (0%)	2/15 (13%)
Any adverse clinical or operational event^e^	3/9 (33%)	1/6 (17%)	4/15 (27%)
Ethics consultation documented	1/9 (11%)	0/6 (0%)	1/15 (7%)
Transfusion medicine notified prior to unit arrival	1/9 (11%)	0/6 (0%)	1/15 (7%)
Surrogate decision‐making	9/9 (100%)	1/6 (17%)^f^	10/15 (67%)

^a^ Two patients had directed units received at the blood bank but none transfused to them (total 5 units released to general inventory). One was a pediatric patient with congenital heart disease admitted for elective cardiac surgery; 2 RBC units were collected from family donors but were not transfused to the intended recipient and were released to general inventory. The other was a young adult with congenital heart disease admitted for elective cardiac surgery; 3 RBC units were collected from family donors but were not transfused to the intended recipient and were released to general inventory. Both cases were elective, and neither patient experienced documented clinical deterioration.

^b^ Patient 1 (adolescent): PICU admission for multiorgan dysfunction in the setting of critical illness; hemoglobin declined to 5.9 g/dL with symptoms while awaiting directed units due to surrogate refusal of standard products; subsequently transfused at hemoglobin 9.2 g/dL to prevent unit outdating, representing a deviation from institutional transfusion guidelines.

^c^ Patient 6 (adult): hemorrhage in the setting of an oncologic diagnosis; hemoglobin declined to 3.6 g/dL with hemorrhagic shock due to refusal of standard products while awaiting directed units.

^d^ Patient 4 (young child): congenital heart disease, elective cardiac surgery delayed pending directed unit availability. Patient 8 (adolescent): congenital condition requiring elective cardiovascular surgery, canceled and rescheduled due to insufficient directed product availability.

^e^ Defined as clinical deterioration, guideline‐deviating transfusion, and/or surgical delay/cancellation. Patient 1 had both clinical deterioration and a guideline‐deviating transfusion; counted once at the patient level.

^f^ Surrogate involvement was not systematically captured for adult patients; one adult request was explicitly documented as a surrogate request, but no documentation was identified for the other adult patients.

The 13 transfused patients had a median age of 17 years (range 0.33–73), with 8 (62%) classified as pediatric. Nine were female; four were male. Eleven (85%) patients were inpatient at the time of transfusion and 2 (15%) were outpatient. Nine (69%) patients underwent transfusion in the elective setting; four (31%) were urgent or emergent. Clinical scenarios included surgeries for congenital heart disease, transplant and oncologic care, major orthopedic surgery, and critical illness. All cases involved refusal of standard inventory unless blood was perceived to be “unvaccinated/safe.” Twelve patients received units exclusively from family members; one received units from both related and non‐related individuals.

Despite the clinical and ethical complexity of these requests, documented oversight among cases that resulted in directed units arriving to the VUMC blood bank was limited: an ethics consultation was documented in one of 15 cases (7%), and transfusion medicine was notified prior to unit arrival in one of 15 cases (7%), after the request had already been approved by the perioperative service.

Four transfused patients (31%) had explicitly documented clinical impacts. Two experienced clinical deterioration in the setting of refusing standard blood products. In one case, hemoglobin declined to 5.9 g/dL with symptomatic anemia while transfusion was delayed awaiting directed units. This patient later received a transfusion at hemoglobin 9.2 g/dL—deviating from institutional guidelines—because the team did not want the unit wasted. In the second case, a patient developed hemodynamic shock with hemoglobin nadir of 3.6 g/dL. Two additional patients experienced surgical delay or cancellation with subsequent rescheduling, attributed to coordinating directed component availability.

## 4 DISCUSSION

In this series, all directed donor units received at VUMC in 2024 and 2025 were motivated by requests for “unvaccinated blood” per documentation in the medical record and associated with documented clinical deterioration, care disruption, and resource inefficiency. These cases highlight a systems vulnerability whereby routing requests directly to an online directed‐donation workflow may bypass standardized counseling and escalation pathways recommended in published guidance.^8^, ^19^

### 4.1 Comparison with structured intervention programs

Our findings contrast with reports from institutions that use structured pathways for vaccine‐related transfusion concerns. At Mayo Clinic, a shared decision‐making approach within a Bloodless Medicine and Surgery Program resulted in most patients ultimately accepting standard blood products, and the institution subsequently restricted directed donation in the absence of medical indication.^17^ Similarly, at Seattle Children's Hospital, structured consultation involving transfusion medicine and ethics supported families in ultimately accepting standard inventory products in pediatric cardiac surgery cases.^21^

Our series reflects a different institutional reality. Most directed donation requests at our institution are routed from the patient's primary medical team or perioperative clinic directly to the blood center's online form without routine escalation to transfusion medicine, resulting in 15 patients proceeding to directed donation with multiple downstream impacts. However, because our case‐finding strategy captures only requests that culminated in unit arrival, we cannot determine how often counseling occurred upstream for requests that were resolved before any directed unit was collected or delivered.

Our cohort included urgent and emergent cases and a majority of pediatric patients, as 9 of 15 patients (60%) and 8 of 13 transfused patients (62%) were under 18 years of age. This observation carries distinct ethical weight. Unlike autonomous adult refusals of standard care, these represent surrogate decisions made by parents on behalf of patients who cannot consent, a critical distinction for ethics committees evaluating such requests. The predominance of pediatric patients may reflect heightened parental anxiety about perceived blood safety, particularly in clinical contexts such as congenital heart surgery where transfusion is nearly inevitable, and warrants further investigation given that parental directed donations carry the highest infectious disease marker positivity rates.^5^, ^26^

Moreover, the clinical deterioration observed in our series, including hemodynamic shock and severe symptomatic anemia while awaiting directed units, challenges claims by some groups that non‐medically indicated directed donation is a benign or low‐risk accommodation. The case in which hemoglobin declined to 5.9 g/dL while awaiting directed units, followed by transfusion at hemoglobin 9.2 g/dL to avoid unit expiration—deviating from institutional guidelines—illustrates how these requests can distort clinical decision‐making in both directions, causing harm from both delay and unnecessary transfusion in a single patient. Ethics committees may be unaware that patients are declining standard products to the point of life‐threatening anemia; these outcome data should inform prospective risk–benefit assessments for future requests. Taken together, these experiences underscore the critical need for standardized institutional processes that include reliable involvement of transfusion medicine expertise and ethics consultation when ethically complex refusals or non‐medically indicated donor requests are present.

### 4.2 Blood center policy variation

A critical systemic factor enabling the cases we observed is heterogeneity in blood center policies. Some blood centers now require documented medical indication for directed donation. For example, Carter BloodCare, a major regional blood supplier, requires that the patient's physician document a medical reason and explicitly excludes directed donations for donor preference and other non‐medical reasons.^27^ In contrast, other blood centers maintain online request forms that can be completed by patients, family members, or clinical staff without any screening for medical indication.

This policy variation creates a critical expertise gap. When a surgeon, nurse, or perioperative staff member assists a patient or family in completing an online directed donation form—as appears to have occurred in many of our cases—the request bypasses transfusion medicine experts who possess specialized knowledge regarding transfusion safety, infectious disease risks in directed donation, and evidence‐based alternatives.

### 4.3 Risks specific to directed donation

Despite being framed as “safer,” directed donations may paradoxically increase risk. Directed units, particularly from family members, have been associated with higher infectious disease marker positivity than volunteer donations.^5^, ^25^, ^26^ First‐time parental donors exhibit infectious disease marker positivity rates of 6–7%, substantially higher than community donors.^5^, ^26^ Although units with reactive infectious disease screening tests are not released for transfusion, these findings remain relevant because directed donors are often first‐time or family donors, groups associated with less favorable infectious‐risk profiles than repeat volunteer donors. These elevated rates likely reflect social pressure within patient‐related donor networks that may compromise the integrity of donor screening. In the parent–child setting, donors may underreport high‐risk behaviors out of a mistaken belief that their blood could not harm their child.^6^, ^19^, ^26^ Contemporary testing has reduced residual transfusion‐transmitted viral risk to very low levels, but not to zero, and first‐time donors are estimated to carry a several‐fold higher residual risk of transfusion‐transmitted infection than repeat donors.^15^, ^28^, ^29^ Notably, all transfused patients in our series received at least one product from a family member, making these concerns especially relevant in our cohort.

Furthermore, directed donations from biological relatives also require irradiation to prevent transfusion‐associated graft‐versus‐host disease (TA‐GVHD), a potentially fatal complication. This mandatory processing step adds operational complexity and cost that clinical teams initiating directed donation requests outside transfusion medicine oversight may not anticipate, and failure to irradiate a biologically related donor unit could have catastrophic consequences. The predominance of family donors in our cohort thus compounds infectious disease risk arising from social pressure on donor screening with operational risk inherent in bypassing standard transfusion safety workflows.

Beyond these immediate hazards, directed donations from close relatives carry immunologic consequences that non‐transfusion medicine clinicians may not recognize. Recipients may form human leukocyte antigen (HLA) antibodies against a family donor, potentially precluding that individual as a future stem cell or organ donor, a particularly consequential outcome in pediatric patients for whom related‐donor transplantation may later become necessary. The risk is further heightened when mothers donate to their children. During pregnancy, mothers commonly develop HLA antibodies against paternally inherited fetal antigens; transfusing maternal blood to the child therefore carries an elevated risk of transfusion‐related acute lung injury (TRALI), a serious and potentially fatal reaction. Without transfusion medicine involvement in directed donation discussions, these risks may go unappreciated until harm has occurred.

In addition to the risks to the intended recipient, the disposition of unused directed units raises distinct concerns. Although 12 RBC units (25% of all directed units received) were released to the general allogeneic inventory, ostensibly mitigating waste, this disposition does not represent equivalent resource recovery. These units originated from directed donors, predominantly first‐time family members, a population with elevated infectious disease marker positivity rates. Once crossed over, however, these units become indistinguishable from standard volunteer community donor products to the ordering clinician and receiving patient. The downstream recipient has no knowledge that the unit they received bypassed the foundational safety layer of voluntary, anonymous donation free from social coercion, nor did they consent to receive such a unit. While infectious disease testing is highly effective, the safety of the blood supply is predicated on layered risk reduction in which no single layer, including testing, is assumed to be infallible. Releasing directed units into the general supply thus does not recoup inefficiency; rather, it transfers risk from the requesting family's unused product to an uninvolved third party, raising concerns about population‐level nonmaleficence that extend beyond the index patient.

### 4.4 Practical implications for health systems

**FIGURE 2:**
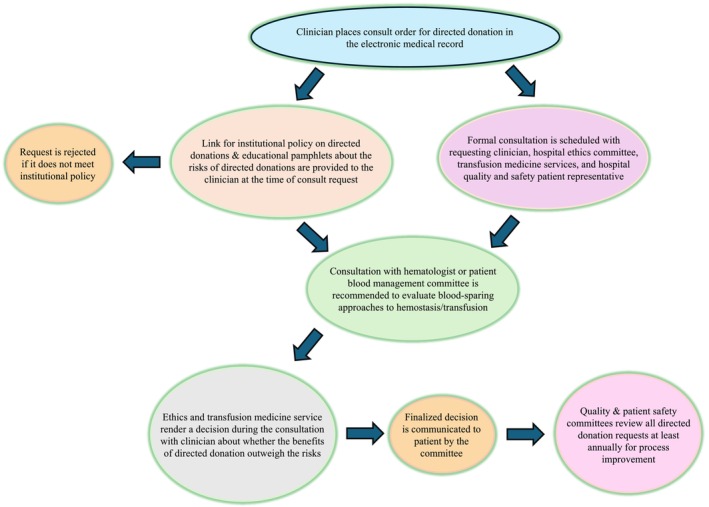
Proposed algorithm to approach requests for directed donations of blood products.

Building on the Mayo Clinic and Seattle Children's experience and established ethical frameworks for directed donation,^6^, ^17^, ^19^, ^21^ health systems should consider several key interventions (Figure 2): (1) mandatory transfusion medicine consultation for all directed donation requests before blood collection proceeds, replacing optional online submission pathways; (2) dedicated bloodless medicine specialists or patient blood management programs with expertise in shared decision‐making; (3) explicit institutional policies prohibiting directed donation solely for donor vaccination concerns or other donor characteristics irrelevant to transfusion safety (e.g., race, ethnicity); and (4) differentiated pathways for urgent, emergent, and pediatric cases given the heightened clinical and ethical stakes in these populations.

Such policies also serve a protective function for clinicians, who may face sustained pressure from patients or families to accommodate non‐medically indicated requests. A clear institutional policy removes the burden of individual refusal and provides a framework for redirecting these conversations to transfusion medicine and ethics for shared decision‐making with appropriate expertise.

Educational materials should explicitly address the false perception that directed donation is safer than standard inventory, emphasizing that COVID‐19 vaccination status is not a medically relevant blood safety attribute and that directed donations based on such characteristics lack scientific support but carry unnecessary risk.^8^, ^9^, ^10^, ^11^, ^19^, ^22^ In addition, some blood centers may charge supplemental per‐unit fees for directed donations (personal communication, 9 February 2026), which may partially offset collection and processing costs but do not eliminate the downstream operational and financial burdens borne by hospital transfusion services. These burdens may include product modifications, separate inventory management and storage, and workflow changes related to issuance.^30^, ^31^ More than one‐third of directed units received by our blood bank were not transfused to the intended recipient, illustrating the resource inefficiency inherent in these requests. Moreover, the release of unused directed units to general inventory should not be interpreted as efficient utilization, as these units carry risk profiles distinct from those of standard volunteer donor products. Health systems should work collaboratively with their blood suppliers to ensure that consultation requirements are implemented upstream, before blood collection proceeds.

## 5 LIMITATIONS

This study has several limitations. The cohort is small and was ascertained from cases in which directed units arrived at the blood bank, not all directed donation requests across the health system. Consequently, we cannot estimate overall request frequency, describe requests resolved through counseling or ethics consultation, or determine the true frequency of such interventions during the study period. Downstream impacts were captured only when documented in clinical notes and likely underestimate the full operational burden, including staff time, coordination efforts, and additional per‐unit costs. Finally, given the descriptive observational design, causal attribution between refusal of standard products and clinical outcomes cannot be definitively established.

## 6 CONCLUSION

Directed donation pursued for “unvaccinated” blood concerns occurred across pediatric and adult settings in both elective and urgent clinical scenarios. These requests were associated with clinical deterioration, care delays, and resource inefficiencies when standard inventory products were refused. The near absence of documented ethics and transfusion medicine oversight in these cases represents a critical gap. Standardized institutional pathways aligned with existing professional guidance may mitigate patient‐care risk and operational disruption.

## FUNDING INFORMATION

No funding was received for this study.

## CONFLICT OF INTEREST STATEMENT

All of the authors report no disclosures related to this study.

## Data Availability

The data that support the findings of this study are available on request from the corresponding author. The data are not publicly available due to privacy or ethical restrictions.
